# Editorialets

**Published:** 1908-07

**Authors:** 


### Editorialets.

THE "RED BACK."—The TEXAS MEDICAL JOURNAL begins its twenty-fourth year this month. For nearly a quarter of a century it has been the friend, ally, advocate and champion of organized regular medicine, the uncompromising foe of quacks and quackery. It was a leading factor in the reorganization and upbuilding of our splendid Association. It is recognized everywhere as the strongest, most original, fearless and outspoken "defender of the faith"—*Regular, Legitimate Medicine,* and it stands for, and has ever advocated the best interests of the State Association and its individual members. It is the most talked of and most quoted journal published, and is universally recognized as representative of progressive, aggressive medicine in Texas. Because we now have a splendid State Association journal, which is given you in consideration of your yearly dues (the Association's Society Reporter), which takes the place of the "Transactions," that is no reason why you should not subscribe for the "Red Back," the early and constant champion of Texas medicine. You need it. The best men in Texas, subscribers for twenty odd years, say they can't do without it. *Subscribe now.* Renew for the twenty-fourth round.

---

BARNES UNIVERSITY, MEDICAL DEPARTMENT,
SECRETARY'S OFFICE.

ST. LOUIS, MO., June 15, 1908.

*Dr. F. E. Daniel, Texas Medical Journal, Austin, Texas.*

DEAR DOCTOR: I will be pleased to place our advertisement with you for the three months—July, August and September; one-half page, three insertions.

Assuring you of my warmest regards, and appreciating thoroughly your firm stand in behalf of the best there is in medical education, I am,

Very sincerely,
C. M. AMENT,
Secretary.

---

I LIKE the "Red Back." It is independent, pointed and spicy, and traveling with the vanguard in the reformatory movement of temperance, public hygiene, and kindred subjects.

Truly and fraternally,
ALEXANDER S. GARRETT.

---

IN ALL of my acquaintance with brother physicians I have never met one who is a regular subscriber to your journal that is not a reasonably well-posted doctor. This is more of a compliment to the doctor than to the journal for the physician that takes and reads the "Red Back" and takes and reads others as well. State pride alone should prompt every one in the family of healers to take his home journal.

Truly,
Beckville, Texas. R. F. HARNESBERGER.

---

NEW BOSTON, TEXAS.—Dr. H. A. Burrows writes: "I can't do without it."

THE panic is still on in the lumber district, but guess you had better let the "Red Back" come along.

Yours truly,

Ratcliff, Texas. S. A. COLLOM.

---

REAGAN, TEXAS, May 9, 1908.

*Dr. F. E. Daniel, Texas Medical Journal, Austin, Texas.*

DEAR DOCTOR: I herewith send you postoffice order for $4.00, which you will please place to my credit on account of "Red Back."

Set us up and let me know to what date.

With hearty wishes for your success and the happiness of "Bettie and the baby," I remain,

Fraternally yours,

To January 1, 1911. S. D. DAVIDSON.

---

DR. J. T. O'BAR, of Ledbetter, Texas, is taking a three months' outing, camping, fishing and shooting at Konohassett, in the mountains. He requests the "Red Back" to be sent there—all samee—can't do without it. When he gets back he will have a good story to tell of how big that bass was that broke his hook and got away.

---

DR. J. T. BLACK, of Elroy, Texas, was married July 8th inst. at Austin to Miss Allie May Wedemeyer. No cards; no cake—for ours.

---

THE ROCKWOOD TUBERCULOSIS SANITARIUM.

DANVILLE, INDIANA.

*Parke, Davis & Co., Detroit, Mich.*

GENTLEMEN: We have used Kreso at the Rockwood Tuberculosis Sanitarium for some months as an antiseptic and deodorizer and are well pleased with it. We use it as a wash for woodwork as well as for mopping the floors. The odor of Kreso is pleasing and patients greatly appreciate having it used in the cottages.

The necessity for continuous effort to diminish the infectiveness of institutions is too well appreciated to merit discussion. We merely wish to state that for the purpose of a general wash and deodorizer we have found Kreso more satisfactory than any preparation of the kind we have ever used.

Very respectfully,

T. VICTOR KELNE,

Consulting Bacteriologist.

KRESO.—In the big sanitary movement now so active in Texas, recently inaugurated by our very efficient State Health Officer and pushed by the Association of Texas Health Officers—"Kreso"—a combination of coal tar preparations, will be found a most valuable factor. It is efficient, safe and non-corrosive, as well as inexpensive. It knocks Platt's Chlorides and carbolic acid higher than a kite. Parke, Davis & Co. will be glad to furnish literature on request. They have thousands of testimonials from sanatorium and health officers. I give only one herewith. I beg to add my own personal endorsement. "No family should be without it," and I intend that mine never shall be.

---

NEW SUBSCRIBERS FOR VOLUME XXIV.—Bledsoe, M. F., Rockland Texas; Blailock, W. R., Dallas, Texas; Blasdell & Thomas, Ballinger, Texas; Chambers, R., Fort Worth, Texas; Carey, E. H., Dallas, Texas; Ellis, E. B., Rule, Texas; Farmer, W. C., San Antonio, Texas; Fly, J. M., Leesville, Texas; Goeth, R., San Antonio, Texas; Gustine, N. W., Barstow, Texas; Graves, Amos, Jr., San Antonio, Texas; Hensley, J. W., Dewville, Texas; Hargas, J. W., Cotulla, Texas; Harrison, C. W., Hidalgo, Texas; Hassell & Hassell, San Antonio, Texas; Hull, Theo. F., San Antonio, Texas; Herff, J. B., San Antonio, Texas; Johnson, B. F., Stockdale, Texas; T. J. Jenkins, Daingerfield, Texas; Jackson, R. S., San Antonio, Texas; Loving, J. M., Austin, Texas; Largent, D., San Antonio, Texas; Lincecum, A. L., Louise, Texas; McDaniel, A. C., San Antonio, Texas; McMakin, — —, San Antonio, Texas; McLaughlin, J. W., Jr., Austin, Texas; McGee, D. B., Harlinger, Texas; O'Donaghue, J. A., Portland, Oregon; Robinson, J. D., Center Point, Texas; Richards, B., Evant, Texas; Southers, G. W., Lincoln, Texas; Stern, Rose S., San Antonio, Texas; Strum, Charlotte, San Antonio, Texas; Snodgrass, T. B., Austin, Texas; Wheeler, F. B., Tilden, Texas; Walters, E. A., Fort Worth, Texas.

